# Children and adolescents´ dental treatment in 2001–2013 in the Finnish public dental service

**DOI:** 10.1186/s12903-019-0828-z

**Published:** 2019-07-01

**Authors:** J. Linden, E. Widström, J. Sinkkonen

**Affiliations:** 1 Public Dental Service Lohja, Lohja, Finland; 2 Institute of Clinical Dentistry, Arctic University of Norway, Tromsø, Norway; 3 National Institute for Health and Welfare (THL), Helsinki, Finland; 4 Finland Reaktor ltd, Helsinki, Finland

**Keywords:** Registers, Oral health, Treatment needs, Public dental service, Treatment measures

## Abstract

**Background:**

The Public Dental Service (PDS) in Finland has catered for the overwhelming majority of the young for more than 50 years. They have had examinations, preventive measures and all other necessary treatment free of charge. This study aimed to survey the treatment needs and treatment measures provided for children and adolescents and changes in these during the period 2001–2013.

**Methods:**

Using each person’s unique identifier, data on patients (< 18 years), their oral health (CPI > 2, D + d > 0) and treatment received in the period 2001–2013 were collected retrospectively from municipal databases in five PDS-units covering 320,000 inhabitants. The National Institute for Health and Welfare gave ethical approval. Permission to use local data was received from the Directors in the PDS units. Treatment measures were grouped into 14 categories and patients into three age categories (0–6 years, 7–13 years and 14–17 years). Trend analysis was used to test changes over time.

**Results:**

About 40,000 children and adolescents visited the PDS each year and 2,488,805 treatment measures were provided for them during the entire study period. The proportion of those in need of treatment decreased from 44.4 to 33.2% during the study period. The most common treatment categories were examinations (613,753, 24.7%), orthodontics (499,033, 20.1%), preventive measures (372,473, 15.0%) and restorative treatment (355,325, 14.3%); these made up 74% of all treatment measures. During the study period, statistically highly significant (*p* < 0.001***) increasing trends were found for examinations, anaesthesia and the total number of treatment measures, and a significant (*p* < 0.001***) decreasing trend in restorative treatment were found for all the young. More preventive treatment measures were provided for those not in need of treatment compared with those in need of treatment.

**Conclusion:**

Although children’s oral health had improved and restorative treatment provided had decreased, the total number of treatment measures increased. Healthy children received frequent examinations and high numbers of preventive treatment measures. Targeting treatment according to needs was not satisfactory.

## Background

Dental caries still affects a great number of children and adolescents in spite of the range of caries preventive methods available [1]. Well-organized oral health care provision systems are in place, such as in the Nordic countries, that provide the young with regular and basically free dental care regardless their place of residence [2]. In Finland, the public sector has catered for children’s dental care since the 1950s, when the school dental services started. In 1972, the Primary Health Care Act obliged municipalities to provide annual examinations and all necessary preventive and dental care free of charge to all persons below 18 years of age. Generally this is undertaken all over the country in the Public Dental Service (PDS). Since the 1970s, children and adolescents’ oral health has improved greatly in many countries including Finland. The mean DMFT value for 12-year-olds was 6.9 in 1975, 1.2 in 1991 and in 2000 [3] and 0.9 in 2013 [4]. Correspondingly, the proportion of caries-free children was 1% in 1975, 30% in 1991, 38% in 2000 [3] and 56% in 2013 [4]. A similar improvement has been seen in the other Nordic and most Western European countries [5].

Oral health has become more important, dental care more systematic and less invasive and self-care definitely more common. Other factors such as economic progress and life style changes have also played a role. Along with improvement of the oral health of the young in Finland, it was intended that adults be able to use the subsidised services of the PDS. All age restrictions on access were gradually abolished up to 2002 [2]. However, in 2009, a third (36.4%) of all treatment measures in the PDS were still provided for children and adolescents [6]. Health improvement can be expected to change the treatment provided.

Little research has been published [6–9] on the content of children’s dental care in general and especially in a longitudinal perspective. Overall, developing outcome measures for oral healthcare is still just beginning [10].

The aim of this study was to survey treatment needs and treatment measures provided in the Public Dental Service for children and adolescents under 18 years of age and changes in them during a 13-year period from 2001 to 2013.

## Methods

Five PDS units in southern Finland using a specific electronic patient registration system (WinHit, In Net OY; www.winhit.fi) were asked to participate in the study. Ethical approval for the study was provided by the National Institute for Health and Welfare (THL 1697284289204448) and permission to use the local data by the directors of health services in each PDS unit in 2001. The number of children and adolescents (< 18 years) in the participating PDS units’ catchment areas was in 2001, 55,103 and in 2013, 60,983 persons [11].

Data on all the young people (< 18 years) who had visited the five PDS units during 2001–2013 were collected retrospectively from each municipal database. For each year, the numbers of all patients who had attended for a dental visit and all treatment measures provided by any professional category (dentists, dental hygienists and dental nurses) were extracted from the databases. Registered data on need for basic periodontal and restorative treatment (CPI > 2, D + d > 0) [12] were also collected.

The patients were grouped into three age categories (0–6 years, 7–13 years and 14–17 years). The items of treatment provided were classified into 13 main treatment areas: clinical examinations including complementary examinations (laboratory tests etc.), preventive care (instruction about oral hygiene, dietary advice, fluoride varnish, fissure sealants etc.), periodontics, restorative care (permanent and temporary fillings, crowns made of filling material), endodontics, treatment of temporomandibular disorders (TMD), orthodontics, prosthetics, anaesthesia (local anaesthesia, sedatives, nitrous oxide), emergency treatment, radiology, oral surgery and other treatment (removal of sutures, local medications, certificates etc).

The R 3.3 environment for statistical computing was used for descriptive and inferential analyses.

To separate possible trends in the volumes of treatment categories from annual fluctuations, the log-volumes of categories were modelled as linear over time, allowing for correlated residuals [13]. A similar model was fitted for the total treatment need over time, and for the agreement of treatment need and preventive treatment provided. Note that a linear model on the log-scale is equivalent to a constant proportional change (in percentages) per year (with random fluctuations, the AR residuals). Overall, the models fitted the data well, except for treatment of temporomandibular disorders and prosthetics with very few available years with known volumes. Statistical significance was tested at the level of *p* < 0.05.

## Results

During 2001–2013, the number of children and adolescents who visited the PDS was around 40,000 each year and fluctuated slightly between a minimum 36,263 (in 2006) and a maximum 40,746 (in 2009), Table 1. The number of patients in the two youngest age groups was stable during the study period and the number of 14–17 year old patients increased slightly from 8270 to 9250 (11.9%; Table 1).

**Table 1:** Numbers of the young patients (< 18 years), total and by age group (0–6 years, 7–13 years and 14–17 years) treated in the five PDS units; numbers of those having visited a dentist, dental hygienist and dental nurse and numbers of treatment measures by treatment category provided for them by year and totally during 2001–2013 as well as the change between 2001 and 2013 (%)

Year	2001	2002	2003	2004	2005	2006	2007	2008	2009	2010	2011	2012	2013	Sum (%)	Change from 2001 to 2013
Patients, all and number in the category														Total number of different individuals in age category during the 13 years.	
All (N)	39,682	39,297	40,022	36,316	37,857	36,263	38,216	36,442	40,746	36,809	40,433	37,386	39,517	118,402	-0,4
0–6 year olds(%)	12,918(33%)	12,479(32%)	12,744(33%)	11,505(33%)	12,314(33%)	11,101(31%)	11,817(31%)	11,117(31%)	13,064(33%)	11,836(32%)	12,604(31%)	10,728(29%)	12,025(30%)	66,572	-6,9
7–13 year olds(%)	18,494(47%)	18,488(47%)	18,734(47%)	16,994(47%)	17,307(46%)	16,940(47%)	17,234(45%)	16,519(45%)	18,603(46%)	17,231(47%)	18,350(44%)	17,922(48%)	18,242(46%)	69,835	-1,4
14–17 year olds(%)	8270(21%)	8330(21%)	8544(21%)	7817(22%)	8236(22%)	8222(23%)	9165(24%)	8806(24%)	9079(22%)	7742(21%)	9479(23%)	8736(23%)	9250(23%)	53,919	11,9
Examined(per cent of patients)														Sum	
0–6 year olds(%)	11,258(87%)	11,008(88%)	11,292(88%)	9632(84%)	10,737(87%)	9698(87%)	10,692(90%)	9305(84%)	10,790(83%)	9477(80%)	11,042(87%)	9092(85%)	10,246(85%)	134,269	−9,0
7–13 year olds(%)	14,871(80%)	14,870(80%)	15,192(81%)	13,563(80%)	13,697(79%)	13,604(80%)	13,833(80%)	12,835(77%)	14,117(76%)	11,836(69%)	13,169(72%)	12,073(67%)	12,833(70%)	176,493	−13,7
14–17 year olds(%)	6694(81%)	6726(81%)	6992(82%)	6137(79%)	6488(79%)	6422(78%)	7373(80%)	6732(76%)	7111(78%)	5088(66%)	7111(75%)	5893(67%)	6396(69%)	85,163	−4,5
In need of basic treatment(per cent of the examined)														Sum	
0–6 year olds(%)	1281(11%)	1176(11%)	1208(11%)	1069(11%)	929(9%)	852(9%)	718(7%)	689(8%)	692(6%)	430(5%)	520(5%)	477(5%)	430(4%)	10,471	−66,4
7–13 year olds(%)	8647(58%)	8382(56%)	8983(59%)	796,459%)	7589(55%)	6335(47%)	5733(41%)	5366(43%)	5640(40%)	5466(46%)	5826(44%)	5488(46%)	5884(46%)	87,303	−32,0
14–17 year olds(%)	4644(69%)	4562(68%)	4809(69%)	4125(67%)	4424(68%)	3812(59%)	4185(57%)	3481(57%)	3383(48%)	2715(53%)	3765(53%)	3550(60%)	3481(54%)	50,936	−25,0
Treatment measures														Sum (%)	
All treatment measures	180,967	196,527	195,887	182,910	182,458	169,864	181,623	178,628	204,659	196,513	208,163	199,309	211,297	2,488,805	16,8
0–6 year olds	35,137(19%)	35,125(18%)	35,827(18%)	32,704(18%)	31,390(17%)	28,596(17%)	32,363(18%)	31,244(17%)	37,470(18%)	34,808(18%)	34,474(17%)	30,788(15%)	35,350(17%)	435,276(17%)	0,6
7–13 year olds	105,888(59%)	115,003(59%)	111,940(57%)	103,898(57%)	102,848(56%)	95,354(56%)	97,201(54%)	94,350(553)	108,471(53%)	107,138(55%)	112,350(54%)	110,470(55%)	114,977(54%)	1,379,888(55%)	8,6
14–17 year olds	39,942(22%)	46,399(24%)	48,120(25%)	46,308(25%)	48,220(26%)	45,914(27%)	52,059(29%)	53,034(30%)	58,718(29%)	54,567(28%)	61,339(29%)	58,051(29%)	60,970(29%)	673,641(27%)	52,6
Examinations	45,682(25%)	45,927(23%)	47,117(24%)	42,656(23%)	43,313(24%)	43,827(26%)	48,141(27%)	46,411(26%)	52,956(26%)	45,388(23%)	52,437(25%)	47,671(24%)	52,227(25%)	613,753(25%)	14,3
Orthodontics	37,466(21%)	42,266(22%)	40,594(21%)	40,197(22%)	40,216(22%)	35,169(21%)	32,756(18%)	31,276(18%)	36,816(18%)	40,036(20%)	37,234(18%)	41,196(21%)	43,811(21%)	499,033(20%)	16,9
Preventive care	19,260(11%)	23,683(12%)	30,034(15%)	27,819(15%)	27,463(15%)	24,659(15%)	28,953(16%)	28,949(16%)	32,987(16%)	30,657(16%)	35,901(17%)	29,779(15%)	32,329(15%)	372,473(15%)	67,9
Restorative treatment	39,706(22%)	38,697(20%)	31,840(16%)	27,454(15%)	26,734(15%)	24,478(14%)	27,061(15%)	26,124(15%)	25,946(13%)	24,000(12%)	23,147(11%)	20,946(11%)	19,192(9%)	355,325(14%)	−51,7
Anaesthesia	11,773(7%)	13,479(7%)	14,497(7%)	14,450(8%)	14,308(8%)	13,416(8%)	14,995(8%)	15,224(9%)	16,974(8%)	17,972(9%)	18,042(9%)	17,458(9%)	17,386(8%)	199,974(8%)	47,7
Radiology	8176(5%)	11,199(6%)	11,525(6%)	11,259(6%)	11,651(6%)	10,643(6%)	12,262(7%)	11,781(7%)	12,708(6%)	10,987(6%)	12,542(6%)	12,456(6%)	13,988(7%)	151,177(6%)	71,1
Other treatment	1956(1%)	16,53(1%)	1389(1%)	872(< 1%)	945(1%)	1839(1%)	2601(1%)	3743(2%)	10,000(5%)	10,708(5%)	11,937(6%)	12,610(6%)	14,498(7%)	74,751(3%)	641,2
Emergency treatment	3316(2%)	4896(2%)	5151(3%)	5261(3%)	5418(3%)	5095(3%)	4593(3%)	4927(3%)	5410(3%)	5930(3%)	5953(3%)	6432(3%)	6799(3%)	69,181(3%)	105,0
Oral surgery	5207(3%)	5718(3%)	5619(3%)	5802(3%)	5405(3%)	4864(3%)	4852(3%)	4738(3%)	4921(2%)	5246(3%)	5217(3%)	5187(3%)	5153(2%)	67,929(3%)	−1,0
Periodontics	6241(3%)	6548(3%)	5940(3%)	5060(3%)	4911(3%)	3718(2%)	2846(2%)	2861(2%)	3030(1%)	2496(1%)	2806(1%)	2836(1%)	3079(1%)	52,372(2%)	−50,7
Endodontics	1332(1%)	1563(1%)	1707(1%)	1679(1%)	1727(1%)	1809(1%)	2180(1%)	2190(1%)	2503(1%)	2677(1%)	2576(1%)	2425(1%)	2474(1%)	26,842(1%)	85,7
Treatment of TMD disorders	190(< 1%)	293(< 1%)	262(< 1%)	342(< 1%)	316(< 1%)	274(< 1%)	302(< 1%)	333(< 1%)	334(< 1%)	352(< 1%)	334(< 1%)	288(< 1%)	331(< 1%)	3951(< 1%)	74,2
Prosthetics	662(< 1%)	605(< 1%)	212(< 1%)	59(< 1%)	51(< 1%)	73(< 1%)	81(< 1%)	71(< 1%)	74(< 1%)	64(< 1%)	37(< 1%)	25(< 1%)	30(< 1%)	2044(< 1%)	− 95,5

Altogether 2,488,805 treatment measures were provided for the young during the study period (Table 1). More than half of them (1,379,888; 55.4%) were provided for the 7–13-year-olds, about a third (673,641; 27.1%) for the 14–17- year olds and the rest (435,276; 17.5%) for the 0–6- year olds. During the 13-year study period, the 0–6- year olds had on average 6.5 treatment measures per patient, the 7–13- year olds 19.8 and 14–17- year olds 12.5 treatment measures each, respectively.

Examinations (613,753; 24.7%), orthodontics (499,033; 20.1%), preventive measures (372,473, 15.0%), restorative treatment (355,325; 14.3%) and anaesthesia (199,974; 8.0%) made up 82.0% of all treatment measures during the entire study period. Prosthetics and treatment of TMD disorders were extremely uncommon (Table 1).

**Fig. 1:**
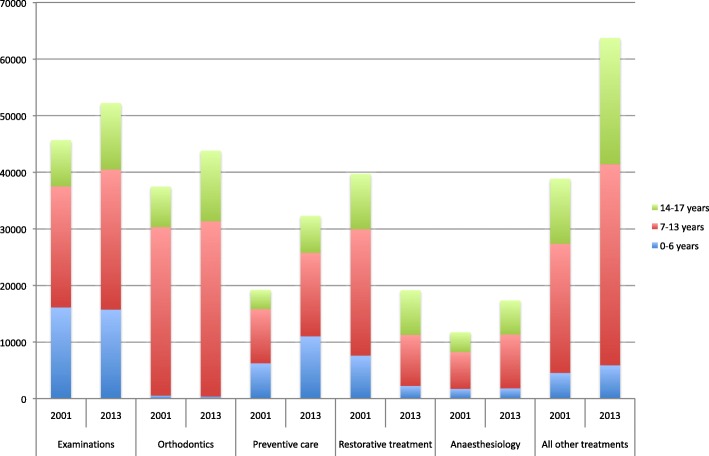
Comparison of numbers of five most common treatment measures (and all other treatments combined) provided for children and adolescents by age group (0–6 years, 7–13 years and 14–17 years) in five PDS units in 2001 and 2013

Differences in the most usual treatment categories between the first and last study year are illustrated in Fig. 1. The total number of treatment measures provided increased by 16.8% from 180,967 to 211,297 (Table 1). Examinations increased from 45,927 to 52,227 (14.3%), orthodontics from 37,466 to 43,811 (16.9%), prevention from 19,260 to 32,329 (67.9%), anaesthesia from 11,773 to 17,386 (47.7%) respectively and restorative treatment decreased from 38,697 to 19,192 (− 57.4%; Table 1, Fig. 1).

**Fig. 2:**
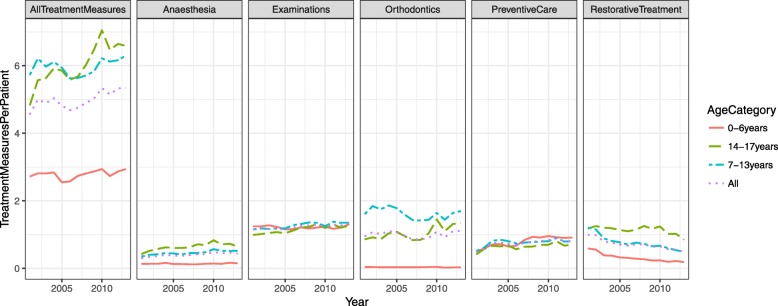
Numbers of the five most usual treatment measures and all treatment measures combined per patient provided for children and adolescents (< 18 years) by age group (0–6 years, 7–13 years and 14–17 years) and totally in five PDS units from 2001 to 2013

The increasing trends in examinations, anaesthesia and the total number of treatment measures, as well as the decreasing trend in restorative treatment were statistically highly significant (*p* < 0.001***) for all the young. Accordingly, there was an increasing trend (from 4.5 to 5.2, *p* = 0.028*) in annual treatment measures per patient (Fig. 2, Table 2). Among the youngest children (0–6 years), the mean number of treatment measures increased slightly (from 2.7 to 2.9, *p* = 0.031*), among the 7–13 year-olds the increase was also small (from 5.7 to 6.3, *p* = 0.320 ^ns^). Among the oldest adolescents (14–17 years), the increasing trend was very clear (from 4.8 to 6.6, *p* = 0.005**).

**Table 2:** Trend analysis on treatment need, on the number of treatment measures per patient in each treatment category provided for all children and adolescents (< 18 years) and separately for the three age categories (0–6 years, 7–13 years and 14–17 years) For the three age categories, only statistically significant treatment categories are presented

Treatment provided	Age category	mu	sd	t	*p*
All treatments and age categories					
All treatments	All the young	0.011	0.004	2.523	0.028^*^
All treatments	0-6 years	0.005	0.005	1.085	0.031^*^
All treatments	7-13 years	0.007	0.006	1.042	0.320
All treatments	14–17 years	0.024	0.007	3.439	0.005^**^
Treatment categories and all ages					
Examinations	All the young	0.012	0.002	5.129	< 0.001^***^
Orthodontics	All the young	0.007	0.014	0.491	0.633
Preventive measures	All the young	0.044	0.029	1.497	0.163
Restorative treatment	All the young	−0.056	0.010	−5.348	< 0.001^***^
Anaesthesia	All the young	0.032	0.007	4.638	< 0.001^***^
Radiology	All the young	0.045	0.029	1.561	0.147
Other Treatment	All the young	0.167	0.104	1.605	0.137
Emergencies	All the young	0.058	0.032	1.785	0.102
Oral surgery	All the young	−0.007	0.006	−1.044	0.319
Periodontics	All the young	−0.059	0.035	−1.652	0.127
Endodontics	All the young	0.052	0.025	2.05	0.065
Treatment of TMJ disorders	All the young	0.030	0.017	1.812	0.097
Prosthetics	All the young	−0.257	0.137	−1.877	0.087
For the three age categories only statistically significant treatment categories are presented.					
Examinations	7-13 years	0.015	0.003	4.246	0.001^***^
Examinations	14–17 years	0.022	0.005	4.612	< 0.001^***^
Orthodontics	14–17 years	0.035	0.015	2.411	0.035^*^
Restorative treatment	0-6 years	−0.092	0.012	−7.933	< 0.001^***^
Restorative treatment	7-13 years	−0.072	0.015	−4.742	< 0.001^***^
Anaesthesia	7-13 years	0.031	0.006	4.719	< 0.001^***^
Emergencies	7-13 years	0.030	0.008	3.836	0.003^**^
Emergencies	14–17 years	0.070	0.019	3.673	0.004^**^
Periodontics	0-6 years	−0.142	0.036	−4.001	0.002^**^
Endodontics	0-6 years	0.024	0.010	2.555	0.027^*^
Endodontics	14–17 years	0.069	0.015	4.608	< 0.001^***^
Prosthetics	14–17 years	−0.149	0.047	−3.15	0.009^**^
Treatment need					
In need of treatment	0-6 years	−0.092	0.010	−9.021	< 0.001^***^
In need of treatment	7-13 years	−0.031	0.019	−1.623	0.133
In need of treatment	14–17 years	−0.035	0.013	−2.808	0.017^*^
Prevention					
No treatment need	0-6 years	0.169	0.055	3.086	0.010^*^
No treatment need	7-13 years	0.054	0.048	1.117	0.288
No treatment need	14–17 years	0.056	0.052	1.086	0.301
In need of treatment	0-6 years	−0.113	0.051	−2.231	0.047^*^
In need of treatment	7-13 years	0.074	0.129	0.571	0.580
In need of treatment	14–17 years	−0.094	0.094	−1.000	0.340

*mu* proportional change (log) per year

*sd* RMSE of the prop, change

*t* the t test statistic for H0 of zero mu

*p* statistical significance

When studying treatment profiles over age categories, in examinations there was an increasing trend in older children (from 1.2 to 1.4, *p* = 0.001**) for 7–13 year olds and for 14–17 year olds (from 1.0 to 1.3, *p* < 0.001***) (Table 2, Fig. 2).

Orthodontic treatment was most common among the 7–13 year-olds and 43.8% of them were treated. They had on average 1.5 treatments per year throughout the study period. Among the oldest children about 27.0% of children had had orthodontic treatment and there was an increasing trend from 0.9 to 1.3 treatment measures (*p* = 0.035*; Fig. 2, Table 2). Orthodontics was uncommon for the youngest children; only 2.9% had had such treatment. During the whole study period, 30.2% of children received orthodontic treatment.

In 2001, preventive treatment was provided for fewer than every second young patient. Among the 0–6 year-olds it almost doubled (*p* < 0.100 ^ns^), among older age groups the increase was less than double (Fig. 2, Table 2).

There were more preventive treatment measures per patient among those not in need of treatment compared with those in need of treatment in every age category. In addition, among those in need of treatment there was a decreasing trend in mean preventive treatment per patient in the 0–6-year-olds (*p* = 0.047*) and 13–17-year-olds (*p* = 0.340 ^ns^; Table 2).

In 2001, there was one restoration for every second child among the 0–6 year-olds and this number had decreased by 69% (*p* < 0.001***) by 2013. Among older children there was one restoration per patient in 2001 and the decline by 2013 was 61% (*p* < 0.001**) for 7–13 year olds and 36% (*p* < 0.320 ^ns^) for 14–17 year olds (Fig. 2, Table 2).

There was one anaesthesia treatment for every tenth 0–6-year-old and one for every second 14–17-year-old. Among the 7–13-year olds there was an increasing trend, from 0.3 to 0.5 treatments (*p* < 0.001***; Fig. 2, Table 2).

During the study period 2001–2013, the proportion of those in need of basic (caries and periodontal) treatment decreased in all age groups: in the 0–6 year olds from 11 to 4% (*p* < 0.001***), in the 7–13 year olds from 58 to 46% (*p* = 0.133 ^ns^) and in the14–17 year olds from 69 to 54% (*p* = 0.017*; Tables 1 and 2).

## Discussion

The main findings in this study were the declining trends in treatment needs and restorative treatment and the increase in the total numbers of treatment measures, especially examinations, anaesthesia and orthodontics in the young (< 18 years). An unexpected finding was that those in need of treatment received less preventive care than those not in need of treatment; the trends diverged in that respect. The fact that more preventive care was provided for those not in need of basic treatment raises noteworthy ethical questions about targeting resources. Are the “at risk” patients being neglected or is restorative treatment still regarded as “the final cure”? In Finland, the recently published evidence-based guidelines for the prevention and management of caries recommend, for caries active children and adolescents, advisory interventions to change unfavourable behaviours, the use of fluoride toothpaste twice a day, fluoride varnish application twice a year, fissure sealants and more specific measures under special circumstances [14]. A recent study of preventive routines in use in children’s and adolescents’ dental care in the PDS in neighbouring Norway showed that clinical practice was not in accordance with the evidence-based guidelines [1]. In Finland, the implementation of the national best practice guidelines depends on how up-to-date the local PDS unit’s strategy is.

“Knowledge translation” is a complex process requiring the leaders of the organisation to introduce the new knowledge and treatment recommendations to the personnel and to make available time and resources to keep up with developments [15]. Unfortunately, this is not everyday practice in dentistry. The trend towards increasing total numbers of treatment measures among the “healthy young” is worrying, especially when politically there has been a desire to improve adults’ access to the PDS. An earlier study showed that the young were five times more likely to be examined in the PDS than adult patients although they were three times less likely to be in need of care [16]. A law enacted in 2011 requires two dental examinations before a child reaches 7 years old and three more examinations before the age of 18 years [17]. The present study recorded more than one examination per patient per year and a trend from 1.2 to 1.3 examinations per patient per year.

The study results show a big (16.9%) increase in orthodontic care provision, which is difficult to explain. There has been no change in the criteria for accessing free of charge orthodontic treatment in the PDS. It is unlikely that bite dysfunctions have increased in the young population but demand probably has increased. In our study, the proportion (30.2%) of children who received orthodontic treatment is in line with reports from the Scandinavian countries [18].

The increase in anaesthesia can be considered to be due to improved pain and fear management. In addition, the treatment is not time consuming [7].

There is little tradition in feasibility evaluation of dental treatment patterns and outcome assessment of dental care [19]. Monitoring of how oral health care functions has focused on economic indicators such as costs and numbers of patients. Local and regional policy-makers, who lack understanding of substance, often have difficulty understanding oral care, so that dentists have had great clinical autonomy [20].

Use of patient management data collected in state-funded dental services or in public and private insurance databases has become a promising method for studying how treatment provided relates to treatment needs and oral health outcomes [21, 22]. In a recent British study, patient management data provided valid accounts of the care without patient recall or selection bias and overall treatment received mirrored treatment needs presented in national epidemiological surveys fairly well [8]. Finland has a long tradition of collecting treatment and performance data in the PDS; these data have however, mainly been used locally, e.g. for paying activity-related remuneration for the dentists and not much for planning of services.

One advantage of this study is that recording of certain oral health indices (treatment needs) and all treatment measures is mandatory in the PDS and, as mentioned, part of each dentist’s salary is based on the treatment measures provided. Thus data from the PDS records have been considered reliable [23]. Further, the data were collected from each unit’s database backup by the same expert using the same script.

A limitation of the study is that the PDS units use many different database systems and produce slightly different data, making it impossible to compare between the units without massive adjustments. Thus, we chose five medium sized or large PDS units in southern Finland using the same patient database system. The total number of Finnish PDS units is 194, most of them are small (< 5000 inhabitants). So, the results of this study can be generalized to medium or large towns in southern Finland. A further limitation of this study is that no information on social background is collected in the PDS register. Also, the information collected on treatment needs and the indicators used were rather crude.

More research is needed on the unexpectedly low use of preventive treatment measures. Also we need more information about the need for and performance of orthodontic care. Almost all the young people in Finland (99%) use the PDS [6]. Throughout the study period, the annual coverage (proportion of the population that attended the PDS) of children and adolescents was high, around 75% [16]. During the study period, the number of dental hygienists doubled (+ 97.9%) and the number of dentists did not change (− 0.0%). When comparing the treatment profile in this study with national data from 2009 it is clear that our figures are in line with national figures and indicate that the selected PDS-units were not outliers among the Finnish PDS units [7]. This study shows that use of routine administrative data collected in the PDS organization can improve transparency of oral health service delivery and offers good tools for leaders and decision makers.

## Conclusions

The volume of treatment need and restorative care decreased in all groups of children and adolescents in the study period. Nevertheless, the total number of treatment measures provided increased. The increase was biggest for examinations, anaesthesia and orthodontics. Children not in need of treatment received more preventive care than those in need of treatment. We conclude that treatment need and treatment provided were not in accordance in children’s oral health care; relatively healthy children received a large amount of examinations and preventive treatment measures.

## Data Availability

The data that support the findings of this study are available from the National Institute for Health and Welfare (THL) but restrictions apply to the availability of these data, which were used under licence for the current study, and are thus not publicly available. Data are, however, available from the authors upon reasonable request and with permission of the National Institute for Health and Welfare (THL) as well as the participating communities.

## Abbreviations

AR residual: Autoregressive residual
CPI: Community Periodontal Index
d: number of decayed decidous teeth
D: Number of permanent decayed teeth
DMFT: Number of decayed, missed and filled teeth
ns: Not significant
PDS: Public Dental Service
THL: The National Institute for Health and Welfare (Terveyden ja Hyvinvoinnin Laitos)
TMD: Temporomandibular disorders
